# Development of an efficient glucosinolate extraction method

**DOI:** 10.1186/s13007-017-0164-8

**Published:** 2017-03-21

**Authors:** T. Doheny-Adams, K. Redeker, V. Kittipol, I. Bancroft, S. E. Hartley

**Affiliations:** Department of Biology, University of York, Wentworth Way, York, YO10 5DD UK

## Abstract

**Background:**

Glucosinolates, anionic sulfur rich secondary metabolites, have been extensively studied because of their occurrence in the agriculturally important brassicaceae and their impact on human and animal health. There is also increasing interest in the biofumigant properties of toxic glucosinolate hydrolysis products as a method to control agricultural pests. Evaluating biofumigation potential requires rapid and accurate quantification of glucosinolates, but current commonly used methods of extraction prior to analysis involve a number of time consuming and hazardous steps; this study aimed to develop an improved method for glucosinolate extraction.

**Results:**

Three methods previously used to extract glucosinolates from brassicaceae tissues, namely extraction in cold methanol, extraction in boiling methanol, and extraction in boiling water were compared across tissue type (root, stem leaf) and four brassicaceae species (*B. juncea*, *S. alba*, *R. sativus*, and *E. sativa*). Cold methanol extraction was shown to perform as well or better than all other tested methods for extraction of glucosinolates with the exception of glucoraphasatin in *R. sativus* shoots. It was also demonstrated that lyophilisation methods, routinely used during extraction to allow tissue disruption, can reduce final glucosinolate concentrations and that extracting from frozen wet tissue samples in cold 80% methanol is more effective.

**Conclusions:**

We present a simplified method for extracting glucosinolates from plant tissues which does not require the use of a freeze drier or boiling methanol, and is therefore less hazardous, and more time and cost effective. The presented method has been shown to have comparable or improved glucosinolate extraction efficiency relative to the commonly used ISO method for major glucosinolates in the Brassicaceae species studied: sinigrin and gluconasturtiin in *B. juncea*; sinalbin, glucotropaeolin, and gluconasturtiin in *S. alba*; glucoraphenin and glucoraphasatin in *R. sativus*; and glucosatavin, glucoerucin and glucoraphanin in *E. sativa*.

**Electronic supplementary material:**

The online version of this article (doi:10.1186/s13007-017-0164-8) contains supplementary material, which is available to authorized users.

## Background

Glucosinolates, B-thioglucoside *N*-hydroxysulfate derivatives, are secondary metabolites found in brassicaceae and related families [1]. Over 120 glucosinolates, which differ in variable aglycone side chains derived from an alpha-amino acid, have been identified and classified into aliphatic, aromatic and indole glucosinolates [2, 3]. Due to their prevalence in cultivated vegetables, spices, oils and animal feed, glucosinolates and their hydrolysis products have been much studied in the context of their effects on human and animal nutrition [4, 5]. Glucosinolates and their breakdown products have also been a focus of studies in dietary prevention of disorders linked to oxidative stress such as cancer and gastric ulcers [2, 6, 7] and more recently, potential undesirable dietary effects such as genotoxicity of glucosinolate breakdown products in broccoli [8] and Pak Choi [9]. The breakdown of glucosinolates has also been studied because of their potential use as agricultural pesticides in a technique known as biofumigation. In biofumigation a glucosinolate-rich crop is mulched into the field, releasing toxic secondary glucosinolate by-products, in order to reduce the incidence of pests, weeds and diseases in the following arable and horticultural crops [10–13].

**Fig. 1:**
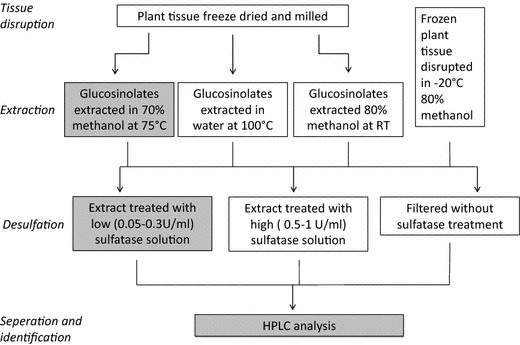
A broad outline of common extraction methods used for glucosinolate analysis. Highlighted in* grey* is the ISO 9167-1 method which was originally intended for glucosinolate extraction from *B. napus* seed but is commonly used for glucosinolate extraction and analysis in all glucosinolate containing plant tissues

Evaluating biofumigation potential requires rapid and accurate quantification of glucosinolates, but current commonly used methods of extraction prior to analysis involve a number of time consuming and potentially hazardous steps. These steps are (1) lyophilisation, or freeze drying, and tissue disruption, (2) extraction in water or methanol, (3) purification of extract, typically by desulfation on DEAE Sephadex, and (4) separation and analysis of (desulfo)glucosinolates. These steps are outlined in Fig. 1 and discussed in more depth below. This study aimed to improve glucosinolate extraction methods by finding alternatives to commonly used steps which are unnecessary or likely to introduce variability.

Myrosinase, an enzyme found in brassicaceae and compartmentalised in cells in close proximity to glucosinolates, is responsible for hydrolysing glucosinolates upon plant tissue disruption. Accurate analysis of glucosinolates therefore requires inactivation of myrosinase prior to tissue disruption. This is achieved by first freezing then freeze drying the tissue which allows disruption by milling or grinding to occur in the absence of water (Fig. 1). Lyophilisation, or freeze drying, is used to remove water from glucosinolate-containing tissues while preventing myrosinase mediated glucosinolate hydrolysis through thermal inhibition. Publications on freeze drying plant tissue have focussed primarily on the production of heat or its implications in generating oxygen sensitive foodstuffs (e.g. space, military or extreme-sport foodstuffs and instant coffee) [14]. To our knowledge, no study has yet examined the efficiency of freeze drying in maintaining glucosinolate concentrations. Freeze drying functions on the principle of sublimation: pressure is reduced below the triple point of water (6.12 mbar, 0.01 °C) at which point sublimation of ice from the sample occurs. The cooling effect of sublimation should be high enough to ensure the sample remains below 0 °C for the initial stage of freeze drying, thus minimizing enzyme-driven glucosinolate hydrolysis. Rapid sample loading and rapid initial pressure drop are also required to avoid sample defrosting before pressure is reduced below 6.12 mbar. Leaves have a high surface area to volume ratio and may defrost quickly, activating myrosinase and reducing final glucosinolate concentration. Despite the importance of the freeze drying process in glucosinolate extraction, many authors do not report details which are likely to affect final concentrations of glucosinolates (e.g. how samples are transported, temperature of the room, whether a heating/cold plate is used and time taken for the pressure to drop).

The most commonly used methods for extraction of glucosinolates from plant material are based on the ISO 9167-1 method [15; highlighted in grey in Fig. 1], which was designed for extraction of glucosinolates from *B. napus* seed and has been adapted to suit the needs of researchers examining glucosinolate profiles of other plant species and tissue types. Although freeze drying is not explicitly detailed in the ISO 9167-1 method, it is an implicit requirement in order to avoid myrosinase mediated glucosinolate hydrolysis during disruption of leaf, stem or root tissues. Once the plant tissue is prepared, the ISO 9167-1 extraction is carried out at 75 °C in 70% methanol for 10 min. Heating the sample is thought to be an essential step to denature myrosinase, thus preventing enzymatic hydrolysis of glucosinolates [16]. Samples are subsequently desulfated by ion exchange chromatography on a DEAE Sephadex column to remove impurities. Desulfoglucosinolates are then separated and identified using HPLC with a reverse phase C18 column and a UV or MS detector. Hazards associated with boiling methanol [17] and the time required for extractions using this method have led researchers to seek alternatives. Replacing heated methanol with boiling water is reported to have comparable [18, 19], and in some cases better [20], extraction efficiencies. Although most glucosinolates are thermostable for the typical 10–30 min heating period, indole glucosinolates such as 4-hydroxy-glucobrassicin and 4-methoxyglucobrassicin have been reported to degrade quickly at temperatures below 100 °C [21]. In addition, prior to 2002 the major glucosinolate in leaves of *E. sativa*, 4-mercaptobutyl glucosinolate, was missed because it self-dimerises via formation of disulphide linkages during extraction [22]. A major challenge therefore to ensuring consistent and repeatable GSL analysis is to create extraction conditions in which myrosinase is inactive, and glucosinolates do not self-react or degrade. A single study, conducted exclusively on radish roots, has demonstrated that cold extraction in 80% methanol does not cause appreciable reduction in glucosinolate concentrations compared to more conventional heated extraction methods [23]. However, myrosinase activity can vary dramatically [24] and whether this method is suitable for extraction of glucosinolates from other glucosinolate containing plants has not previously been assessed.

A desulfation step is often carried out post extraction to purify desulfoglucosinolates and improve accuracy and identification from HPLC. However, the desulfation reaction of glucosinolates can be affected by feedback inhibition of the enzyme which causes incomplete desulfation of glucosinolates [25]. In addition, rhamnopyranosyloxy-benzyl glucosinolates extracted from *M. oleifera* have been shown to be completely converted and degraded by the desulfation purification step [26]. Due to these drawbacks, and the additional time and potential error extra steps can introduce, some authors have skipped the purification and desulfation steps entirely [19, 26, 27] (Fig. 1).

We have tested each stage of glucosinolate analysis from the roots, stems and leaves of *B. juncea*, *S. alba*, *R. sativus*, *E. sativa and B. napus* and suggest a number of adjustments/improvements which can be made to reduce the costs, time and variability associated with glucosinolate analysis. Specifically, this study aims to address the following questions:

1. How do lyophilisation conditions affect glucosinolate concentrations?
2. Is lyophilisation a necessary step for glucosinolate extraction from green tissues?
3. Do extractions in hot methanol, cold methanol and boiling water yield comparable glucosinolate concentrations across a range of brassicaceae species and tissue types?
4. How do desulfation time and enzyme concentration affect final glucosinolate concentrations?
5. Is desulfation a necessary step for glucosinolate extraction from green tissue?

## Methods

### Plant material

*B. napus* used in the freeze drying tests were grown in 1 L pots filled with Terra-green in a controlled temperature glasshouse (regulated from 17.6 to 27.7 °C). At 3–4 weeks post germination, leaves were removed and halved down the limits of the midrib, excluding the midrib from the final sample. Leaf halves were immediately frozen in liquid nitrogen and stored at −80 °C for a maximum of 1 week.

*B. juncea* (cv. ISCI99), *R. sativus* (cv. Bento), *S. alba* (cv. Ida Gold) and *E. sativa* (cv. Nemat) plants were grown by Barworth agriculture ltd. in a sandy loam soil dominated fields (coordinates: 53.000371, −0.290404) from 31/07/2014 to 25/09/2014. Total stem and total leaves were cut from flowering plants and immediately frozen in liquid nitrogen; root samples were gently washed and dried before freezing in liquid nitrogen. Samples were stored at −80 °C for a maximum of 2 months.

### Freeze drying

Samples wrapped loosely in aluminium foil were transported on dry ice and loaded into one of two freeze driers (Table 1). Maximum loading time was 30 s.

**Table 1:** Freeze drier characteristics

Freeze drier	Room temp (°C)	Cooling plate	Time to 5 mbar (s)	Lowest pressure (mbar)	Freezer temperature (°C)	Model
A	22	Yes	90	0.12	−45	Lyotrap, LTE scientific ltd. 1 chamber
B	28	No	65	0.16	−53	Thermo, Heto Powerdry LL3000 4–6 chambers

### Tissue disruption

(i) Freeze dried plant tissue was homogenised to a roughly ground powder (approximately 0.1 cm particle size) using a grinder (Lloytron, E5601BK) Homogenised ground samples were milled at a frequency of 20 Hz for 10 min (Retch, MM400) with 2 steel ball bearings to a fine powder (particle diameter <0.1 mm). Samples were then sealed and stored at 20 °C for up to 9 months.
(ii) Frozen fresh *B. napus* leaf halves (experiment 2, Table 2) were placed in 2 ml eppendorf vials and stored at −20 °C. 1.755 ml of 80% methanol precooled at −20 °C, 25 µl of 5 mM sinigrin and 2 small ball bearings were added. Samples were milled for 10 min at frequency 20 Hz (TissueLyser II, Qiagen). Final concentrations of methanol were estimated by incorporating average leaf moisture content of fresh *B. napus* leaves according to Eq. (1). Final concentration of methanol ranged from 79.3 to 79.9% and leaf moisture content accounted for <1% of final liquid volume.

**Table 2:** Summary of methods used

Experiment	Fig	Species	Tissue	Freeze drying/tissue disruption	Extraction	Desulfation	HPLC
1—Effect of freeze drier on GSL concentration	2	*B. napus*	Leaves	FD-A or FD-B/mill	Cold methanol	0.3 U/ml for 24 h	ISO 9167-1 method
2—Comparison of GSL extraction from freeze dried tissue with extraction from wet tissue	3	*B. napus*	Leaves	FD-A or −20 °C methanol	Cold methanol	0.3 U/ml for 24 H	ISO 9167-1 method
3—Comparison of extraction methods	6, 7	*R. sativus* *B. juncea* *S. alba* *E. sativa*	Leaves, stems, roots	FD-A	Hot methanol, Cold methanol, Boiling water	0.3 U/ml for 24 H	ISO 9167-1 method
4—Comparison of desulfation/purification methods	8, 9	*R. sativus* *B. juncea* *S. alba* *E. sativa*	Leaves, stems, roots	FD-A	Cold methanol	0.3 U/ml for 12, 24, 48 h, and 5 U/ml for 16 h or filtration	ISO 9167-1 method for desulfoGSL, Herzallah and Holly method for intact GSLs

1 $${\text{C}}_{MeOHf} = \frac{{c_{MeOHi} \times V_{MeOHi} }}{{m_{av} \times m_{dl} + V_{MeOHi} }}$$

where c_MeOHf_ is final methanol concentration (%), c_MeOHi_ is initial methanol concentration (90%), V_MeOHi_ is initial methanol volume (1.755 ml), m_av_ is the average moisture content per dry weight (in this case 0.22 ml/g), m_dl_ dry mass of leaf sample (g).

### Glucosinolate extraction

Extractions were carried out in one of three ways (Fig. 1). In each case 50 µl of a 5 mM gluctropaeolin (for *B. juncea* samples) or 20 mM sinigrin (for all other samples) internal standard was added.

#### Hot methanol extraction (based on the ISO 9167-1 method)

0.1 g of plant material was preheated at 75 °C for 3 min in a 20 ml falcon tube. 4.95 ml of 70:30 methanol:water, preheated to 75 °C and the internal standard was added. The sample was incubated at 75 °C for 10 min, and manually shaken every 2 min. The sample was then centrifuged at 4000 rpm (Jouan, model:B 3.11) for 10 min. Supernatent was stored at −20 °C or desulfated directly.

#### Cold methanol extraction (Ishida et al. [23])

5 ml of 80:20 methanol:water at 20 °C was added to 0.1 g plant tissue and the internal standard was added. The sample was shaken and left to stand for 30 min at room temperature. The sample was then mixed at 70 rpm with a platform rocker for a further 30 min (Bibby, STR6) before centrifugation at 4000 rpm (Jouan, model:B 3.11) for 10 min. Supernatent was then filtered through a 0.22 µm syringe filter (Millex GP) for direct injection on HPLC, or unfiltered if applied to Sephadex column in a purification step.

#### Boiling water extraction (adapted from Herzallah and Holley [19])

25 ml of boiling water was added to 0.1 g of freeze dried and milled plant tissue in a 150 ml erlenmeyer flask and the internal standard was added. Sample was heated at 100 °C and stirred with a magnetic stirrer hot plate for 10 min. Sample was heated for a further 4 h at 70 °C before centrifugation at 4000 rpm (Jouan, model:B 3.11) for 10 min. Sample was topped up to 20 ml with deionised water.

### Purification and determination of activity of sulfatase

Sulfatase from Helix pomatia type H-1 (Sigma, S9626) was purified according to Wathalet et al. [25]. 25 mg of sulfatase was added to 1 ml 40% ethanol and centrifuged at 8000 rmp for 1 min (eppendorf centrifuge, 54,151). The supernatant was transferred to a fresh 2 ml eppendorf tube, 1 ml of pure ethanol was added to precipitate the sulfatase before being centrifuged at 8 krmp for 1 min. The supernatant was discarded and the sulfatase pellet air dried and redissolved in 2 ml of water.

Activity of sulfatase was determined based on the ISO 9167-1 method. 1 ml of buffered 0.15 mM sinigrin solution (3 ml of 5 mM sinigrin, adjusted to 100 ml with a solution containing 40 ml 0.2% ethylene diamine, 73 ml 0.2% acetic acid; adjusted to pH 5.8) in a quartz cuvette was placed in a UV spectrometer set to 229 nm. At t = 0, 25 µl of diluted and undiluted purified sulfatase was added to the cuvette and measurements taken over the course of 4 h. The tangent to t = 0 was plotted and its gradient (ΔA/Δt) measured. Activity was calculated using Eq. (2):

2 $${\text{Activity }}\left( {\text{U/ml}} \right) = \frac{\Delta A \times 5.7}{{\Delta tA_{e} }}$$

where ΔA/Δt is the gradient at t = 0 and A_e_ is the difference between absorbance at equilibrium and absorbance at t = 0.

The activity for Sulfatase from Helix pomatia type H-1 (Sigma, S9626) given by the supplier is determined by desulfation of p-nitrocatechol sulfate and is an order of magnitude higher than the activity measured for desulfation of sinigrin using this method.

### Desulfation of glucosinolates

As per the ISO 9067-1 method, columns were prepared with 0.5 ml Sephadex slurry (2 g DEAE Sephadex beads in 30 ml 2 M acetic acid.) and activated with 2 ml imizadole formate (6 M). Columns were washed twice with 1 ml water. The column was washed twice with 1 ml 20 mM sodium acetate (pH 4.0) and 75 µl of purified sulfatase was added (5 or 0.3 U/ml). Columns were incubated at room temperature for either 12, 24 or 48 h before elution of desulfoglucosinolates with two 1 ml volumes of water. For the reduction of disulphide linkages, from dimerized desulfoglucosatavin in *E. sativa* extracts 3 g TCEP (Tris(2-carboxyethyl)phosphine hydrochloride powder Sigma, C4706) was added to 1 ml of desulfated extract. Desulfoglucosinolates were stored at −20 °C before high performance liquid chromatography analysis (Additional file 1).

For the high sulfatase treatment, between 0.5 and 1 ml of sample was added due to insufficient sample volume remaining.

### HPLC

A Waters 600E system controller attached to a Waters 717 autosampler, Waters 996 photodiode array detector and SphereClone 5µ ODS(2) column (Phenomonex) were used for separation and detection of desulfo and intact glucosinolates.

#### HPLC analysis of desulfoglucosinolates—adapted from ISO 9167-1

A reverse phase C18 column (Phenomonex, SphereClone 5µ ODS(2), 150 mm × 4.6 mm) was equilibrated for 30 min with a mobile phase which consisted of 100% diH_2_O. Flow rate was set to 1 ml/min and samples separated according to programme for desulfoglucosinolates detailed in Table 3. Mobile phase solutions were degassed for 30 min in a sonicator (Decon, Sussex England).

**Table 3:** Mobile phase conditions for separation of desulfoglucosinolates

Time	% Solution A	% Solution B	Transition
0	100	0	
30	0	100	Linear gradient
35	0	100	
40	100	0	Linear gradient
50	100	0

- Solution A: 100% diH_2_O
- Solution B: 70:30, diH_2_O:acetonitrile

Desulfoglucosinolates were quantified using 229 nm wavelength within the UV spectrum. The HPLC PDA detector allowed a full spectrum analysis from 180 to 800 nm, allowing comparative UV–visible spectra analysis, which aided in identifying unknown glucosinolates. Through standard injections and HPLC–MS identification we were able to confirm the id’s of these reported glucosinolates. Desulfated purified standards: sinigrin (sigma aldrich), glucotropaeolin, glucoraphenin, glucoraphanin, glucerucin, glucobrassicin, gluconasturtiin, sinalbin, progoitrin and glucoiberin (phytoplan).

#### Mass spectrometry

Major glucosinolates for which no commercial standard is available were identified using an MS detector (Bruker maXis UHR-TOF) with the following settings:

- Source: Standard electrospray (flow split 1/10 from LC)
- Nebulizer: 2.0 bar
- Dry gas: 6.0 L/min
- Dry gas heater: 25 °C
- Capillary voltage: 3500 V
- Ion polarity: positive
- Spectra rate: 1 Hz

#### HPLC analysis of intact glucosinolates—adapted from Herzallah and Holly [19]

A C18 column (Phenomonex, SphereClone 5μ ODS(2)) was equilibrated for 3 h with a mobile phase which consisted of 80 mL (0.02 M) TBA (tetrabutylammonium bromide) and 20 mL ACN (acetonitrile) with detection at 229 nm. The flow rate was set at 1.0 ml/min and separated according to programme for desulfoglucosinolates detailed in Table 3.

- Solution A: 100% TBA (0.02 M)
- Solution B: 70:30, TBA (0.02 M):acetonitrile

Glucosinolates were quantified using the chromatogram from 229 nm and standard curves were constructed using pure sinigrin (sigma aldrich), glucotropaeolin, glucoraphenin, glucoraphanin, glucerucin, glucobrassicin, gluconasturtiin, sinalbin, progoitrin and glucoiberin (phytoplan).

In the case of glucoraphasatin in *R. sativus* leaves and glucotropaeolin in *B. juncea* minor alterations were made to avoid peaks co-eluting. The mobile phase programme for *R. sativus* leaves was 100% A for 5 min, followed by a 35 min linear gradient to 66% B followed by a 5 min linear gradient to 100% B followed by a 5 min linear gradient to 100% A. For *B. juncea* leaves, an isocratic 85:15, TBA (0.02 M):acetonitrile mobile phase for 70 min was used.

### Determination of myrosinase activity

Activity of pure myrosinase was tested in water and 80% methanol solutions containing 0.25 mM sinigrin and 0.1 mM ascorbic acid, a myrosinase cofactor [30]. Myrosinase was added at t = 0 and absorbance of sinigrin at 229 nm was measured over the course of an hour. Activity was measured at room temperature (25 °C).

### Determination of glucosinolate thermostability

A 50 µl of 10 mM sinigrin, 10 mM glucotropaeolin, 10 mM glucobrassicin solution was added to 0.95 ml water or 70% methanol preheated to 100 or 75 °C respectively and sealed in 1.5 ml eppendorf tubes. Samples were maintained at either 100 or 75 °C for 5, 10, 30 and 60 min and intact glucosinolate concentrations analysed with HPLC following the adapted Herzallah and Holly method [19].

### Calculation of glucosinolate content

Glucosinolate content, expressed in µmol/g were calculated according to the ISO 9067-1 method (Eq. 3):

3 $$Glucosinolate\,content = \frac{{A_{g} }}{{A_{s} }} \times \frac{n}{m} \times K_{g} \times \frac{100}{100 - w}$$

where A_g_ is the peak area corresponding to desulfoglucosinolate; A_s_ is the peak area corresponding to internal standard; n is the quantity, in micromoles, of the internal standard; m is the mass of the test portion; K_g_ is the response factor of the desulfoglucosinolate relative to the internal standard; w is the moisture and volatile matter content, expressed as a percentage by mass of the test sample.

### Statistical analysis

Paired two tailed *t* test analysis were carried out on total *B. napus* glucosinolate content per leaf half in experiments 1 and 2 with Microsoft excel (Table 2). For determination of significance of effect of method on final glucosinolate content estimates in experiments 3 and 4 (Table 2), repeat measure ANOVA analyses were carried out for each glucosinolate with R statistical software package (version 3.3.1).

## Results and discussion

### Lyophilisation

Modifications to the ISO9167-1 method (specifically created for the extraction and analysis of glucosinolates from oil rape seed samples) are required for analysis of plant green tissues (leaves, stems and roots). A number of prior-to-analysis steps, such as sampling in the field, cleaning (if required), freezing, crushing, storage or/and shipping and reduction of sample amount have been discussed by Wathelet et al. [28] and are not revisited here. These preliminary steps are followed by lyophilisation, or freeze drying, to remove water from glucosinolate containing tissues while preventing myrosinase mediated glucosinolate hydrolysis through thermal inhibition. This process allows subsequent tissue disruption without risking glucosinolate degradation.

**Fig. 2:**
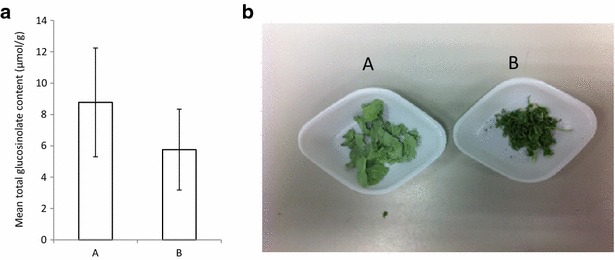
**a** Total glucosinolate concentration of *B. napus* leaf halves dried in freeze drier *B* are significantly lower (paired *t* test, *p* = 0.009) than leaf halves dried in freeze drier *A*; **b** *B. napus* leaf tissue dried with freeze drier *B* is deformed and *darker Error bars* represent standard error

We tested reproducibility of glucosinolate concentrations extracted after lyophilisation in separate freeze driers (Table 4). Fresh *B. napus* leaves were halved, loosely wrapped in foil, flash frozen in liquid nitrogen and transported in dry ice to be dried in separate freeze driers (Table 4). Total glucosinolate concentrations were significantly higher in samples dried in freeze drier A than freeze drier B (Fig. 2a). In addition, samples dried in freeze drier B developed a darker hue and deformed more than samples in dried in freeze drier A (Fig. 2b). Plant tissue samples have been shown to deform during the freeze drying process when temperatures exceed the glass transition state and melting point of water [29]. It is likely that samples placed into freeze drier B may have defrosted before the pressure had reduced below the 6.12 mbar required for sublimation due to higher temperatures and the lack of cooling plate. As a result, enzyme mediated hydrolysis of glucosinolates may have occurred at the initial stage. Additionally, as sublimation slows over time due to the remaining water vapour passing through a dry layer of increasing thickness and because water is increasingly more tissue bound, the sample temperature may have increased to above 0 °C in freeze drier B, causing defrosting.

**Table 4:** Freeze drier characteristics

Freeze drier	Room temp (°C)	Cooling plate	Time to 5 mbar (s)	Lowest pressure (mbar)	Freezer temperature (°C)
A	22	Yes	90	0.12	−45
B	28	No	65	0.16	−53

These results underline the need for a more substantive study to assess optimal conditions for freeze drying plant tissues for glucosinolate analysis. It is clear that differences in freeze drying can introduce significant variability in retained glucosinolate concentrations (Fig. 2a).

A cold methanol extraction method may be sufficient to (1) inactivate myrosinase and (2) efficiently extract glucosinolates, precluding the need for the lyophilisation step altogether. We tested this by comparing glucosinolates extracted from one half of a *B. napus* leaf in 80% methanol without freeze drying against glucosinolates extracted from the other half, first dried in freeze drier A and then extracted using the cold methanol extraction method.

**Fig. 3:**
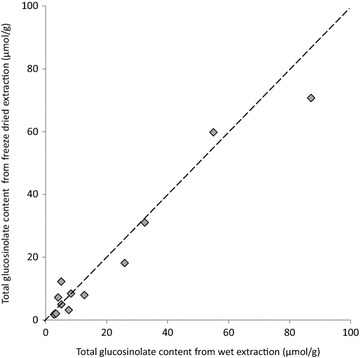
There is no difference in final glucosinolate concentrations between freeze drying or direct extraction in −20 °C methanol. *B. napus* leaves were cut in half and frozen. One half was freeze dried prior to glucosinolate extraction, the other half was extracted directly into −20 °C methanol (n = 12; paired *t* test, *p* = 0.15; R^2^ = 0.96). The *dashed line* represents equivalence of x and y

No significant difference in final glucosinolate concentration was found between the two methods (Fig. 3). Freeze drying is an energy intensive and costly process requiring long drying times under continuous vacuum and the significant effect of freeze drier parameters on final glucosinolate concentrations (Fig. 2a) highlights a potential source of variation between studies. If long term storage of plant tissue samples is not required, skipping the freeze drying step and extracting glucosinolates directly into cold methanol (−20 °C) is cheaper, quicker and less hazardous.

## Extraction

**Fig. 4:**
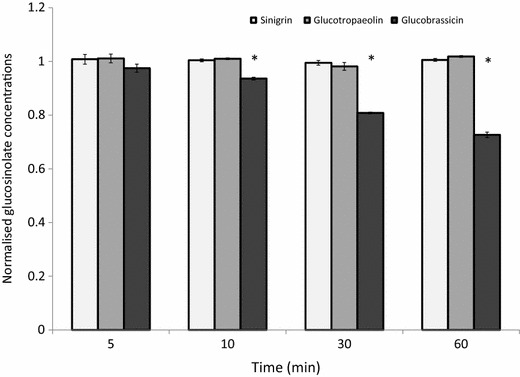
Concentrations of representative aliphatic (sinigrin) and aromatic (glucotropaeolin) glucosinolates were not reduced over the course of an hour at 100 °C. The representative indole glucosinolate (glucobrassicin) is degraded at 100 °C.* Asterisks* represent significant difference from concentration at t = 0 (paired *t* test, *p* < 0.05)

Some authors have highlighted that glucosinolates, specifically indole glucosinolates, are heat sensitive and are significantly degraded in temperatures ≥75 °C in <10 min [21]. This has serious implications for accuracy and reliability of the ISO 9167-1 extraction method, which recommends extractions occur in boiling 70% methanol (75 °C) for 10 min, as well as the less commonly used boiling water extraction (100 °C). In order to first test whether thermal degradation of glucosinolates was likely to occur with these methods we measured the glucosinolate concentrations of pure sinigrin (aliphatic), glucotropaeolin (aromatic) and glucobrassicin (indole) in boiling water (Fig. 4) and boiling 70% methanol (data not shown). Sinigrin and glucotropaeolin did not significantly decrease over 60 min suggesting that extraction in boiling water or methanol is unlikely to affect the concentrations of these glucosinolates. However, glucobrassicin was thermally degraded at 100 °C and data from extractions carried out at these temperatures or above (such as with microwave based methods) may underestimate the concentration of glucobrassicin and other indole glucosinolates. Boiling an extract in water for 10 min degrades glucobrassicin by an estimated 7%.

**Fig. 5:**
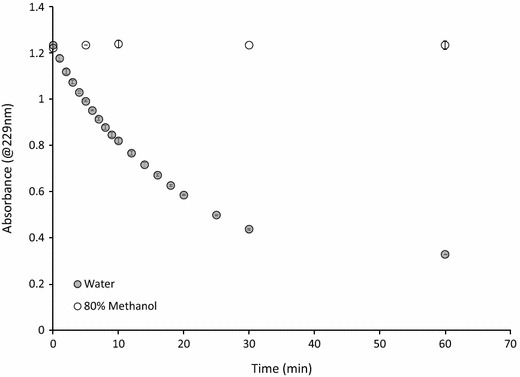
Spectrophotometric analysis of sinigrin hydrolysis kinetics in water and 80% methanol (*n* = 3) by purified myrosinase (0.05 mg/ml) at room temperature (25 °C)

Activity of pure myrosinase was tested at 25 °C in water and 80% methanol solutions containing 0.25 mM sinigrin and 0.1 mM ascorbic acid, a myrosinase cofactor [30]. Absorbance of sinigrin at 229 nm, at room temperature (25 °C), was measured over the course of an hour after myrosinase addition. Myrosinase was inactive in 80% methanol (Fig. 5) suggesting that heating methanol at 75 °C for 10 min in order to inactivate myrosinase may be an unnecessary step for extracting glucosinolates from plant tissue.

Glucosinolates from *B. juncea*, *S. alba*, *R. sativus* and *E. sativa* leaves, stems and roots were extracted (1) in boiling water for 10 min followed by a 4 h incubation at 70 °C, (2) in 70% methanol at 75 °C, or (3) in 80% methanol at room temperature (~20 °C) for 30 min standing followed by 30 min shaking at 70 rpm. All extracts were centrifuged and desulfated with sulfatase according to the ISO 9167-1 method. Major glucosinolates from these species can be found in Table 5.

**Table 5:** Glucosinolates examined in this study

Common name	Chemical name	Structure	Species, tissue type
Sinigrin	2-Propenyl	Aliphatic	*B. juncea* L, S, R
Glucoraphenin	4-Methylsulfinyl-3-butenyl	Aliphatic	*R. sativus* L, S, R
Glucoraphanin	4-Methylsulfinylbutyl	Aliphatic	*E. sativa* L, S, R
Glucosatavin	Mercaptobutyl	Aliphatic	*E. sativa* L, S, R
Glucoraphasatin or hydroxyglucoerucin	4-Methylthio-3-butenyl	Aliphatic	*R. sativus* L, S, R
Glucoerucin	Methylthiobutyl	Aliphatic	*E. sativa* S, R *S. alba*, R
Sinalbin	4-Hydroxybenzyl	Aromatic	*S. alba* L, S, R
Glucotropaeolin	Benzyl	Aromatic	*S. alba* L, S, R
Gluconasturtiin	Phenylethyl	Aromatic	*B. juncea* R *S. alba* R
Methoxyglucobrassicin	4-Methoxy-3-indolylmethyl	Indole	*S. alba* R

L, S and R correspond to leaf, stem and root respectively. Letters in underline represent major glucosinolates of those tissues (>10 µmol/g dry weight)

**Fig. 6:**
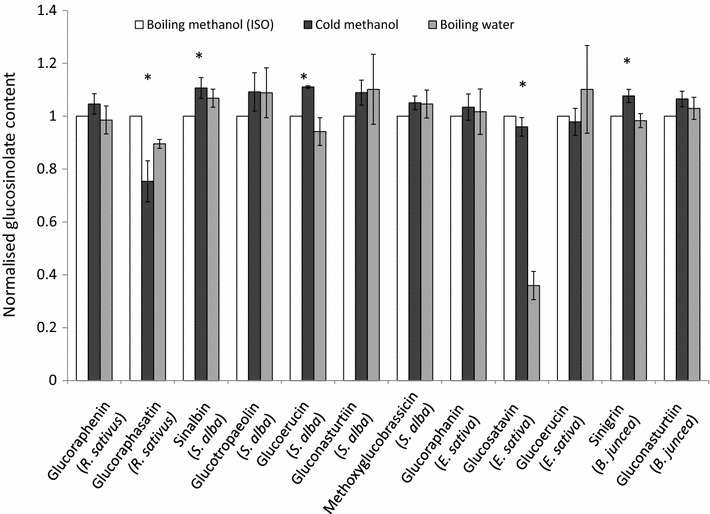
Extraction of glucosinolates (≥1 µmol/g) in plant tissues across the three extraction methods. Glucosinolate concentrations from the cold methanol and boiling water extraction methods are normalised to the glucosinolate concentrations obtained from the ISO9167-1 (75 °C methanol) method (n = 4–12). *Error bars* represent standard error. *Asterisks* represent a significant effect of extraction method on glucosinolate concentration (repeat measure ANOVA, *p* < 0.05)

**Fig. 7:**
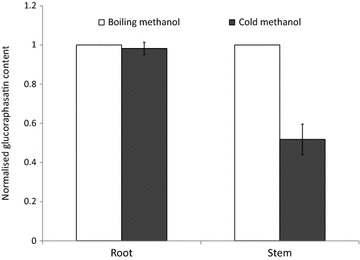
The cold extraction method yields less glucoraphasatin in *R. sativus* stems relative to the ISO 9167-1 (boiling methanol) extraction method (n = 4). Values normalised to the ISO method results. *Error bars* represent standard error. *Asterisks* represent significant difference from the ISO 9167-1 (boiling methanol) method (paired *t* test, *p* < 0.05)

Figure 6 compares glucosinolate concentrations obtained using the cold methanol method and boiling water method normalised against the ISO 9167-1 boiling methanol method. For most glucosinolates, across most tissue types and species, the three extraction methods yield similar results. We found that extraction with cold methanol produced a significantly higher estimated concentration of sinalbin in *S. alba* and sinigrin in *B. juncea* than the hot methanol extraction (Fig. 6). Surprisingly, given the sensitivity of glucobrassicin to thermal degradation (Fig. 4), extraction in boiling water did not significantly reduce the concentration of the indole glucosinolate: methoxyglucobrassicin relative to the other two methods. However, glucosatavin was extracted with lower efficiency from leaves of *E. sativa* using the boiling water method (Fig. 6). It seems unlikely that this glucosinolate is less thermostable than other glucosinolates and was therefore degraded by the extraction method since reduced extraction efficiencies are not observed for stem and root samples. There are no published explanations or hypotheses that might help to explain the observed lower extraction efficiencies for glucosatavin using the boiling water method. Glucoraphasatin extraction using cold methanol appears to be significantly less effective than the standard ISO method (Fig. 6), however this was driven by poor extraction efficiencies from *R. sativus* stems (Fig. 7). Ishida et al. reported a significant 5% increase in glucoraphasatin concentrations extracted from *R. sativus* roots using the cold methanol method [23]. In this study, extraction efficiencies of glucoraphenin in *R. sativus* roots with a cold methanol method were comparable to extraction efficiencies using the boiling methanol method (Fig. 7).

No glucosinolates were detected in a subset of samples extracted in cold water indicating the presence of active myrosinase leading to their degradation (data not shown). However, the cold methanol extraction did not significantly affect the concentration of the internal standard relative to the boiling methanol method (data not shown), providing additional evidence that myrosinase is inactivated in 80% methanol without heating (Fig. 5).

These data demonstrate that 80% cold methanol can be used instead of boiling methanol to extract glucosinolates across a broad spectrum of brassicaceae species and tissue types. With the exception of glucoraphasatin in *R. sativus* shoots, replacing hot 70% methanol with cold 80% methanol did not significantly reduce glucosinolate concentrations, yet marginally increased recovery of sinalbin in *S. alba* and sinigrin in *B. juncea*. It is advised, due to reduction in steps and hazard as well as improved or comparable glucosinolate recovery, that a cold methanol extraction is used instead of a boiling methanol extraction for most glucosinolate containing green tissues.

### Purification

**Fig. 8:**
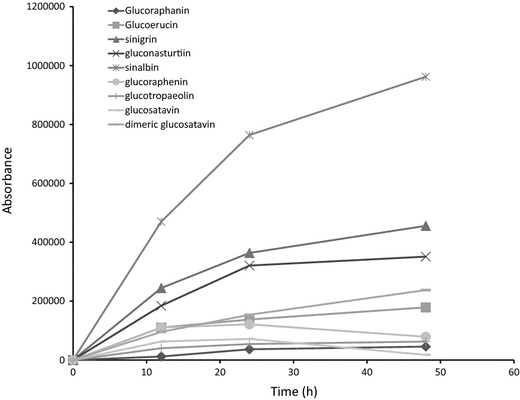
Absorbance values for representative desulfoglucosinolate extracts from *B. juncea*, *S. alba*, *R. sativus* and *E. sativa* extracts treated with sulfatase solution for 12, 24 or 48 h. These values are reflective of desulfoglucosinolate recovery and not the initial glucosinolate concentration

Purification of extract according to the ISO 9167-1 method is carried out by introducing 1 ml of extract to a column containing 0.5 ml of Sephadex solution. The column is rinsed with a 20 mM acetate buffer at pH 4.0 to avoid possible reduction of indole glucosinolates recovery [28]. 75 µl of sulfatase solution with an activity above 0.05 U/ml is applied and left to act overnight. We tested the extraction efficiency of the ISO 9167-1 purification step at the described pH 4.0, at 20 °C for 12, 24 and 48 h. Complete desulfation of glucosinolates in rapeseed extract required a minimum of 11 h in operating conditions of 30 °C and pH 5.8 [25] so it was expected that an overnight 12 h desulfation period may be insufficient for complete desulfation of samples at room temperature. Figure 8 shows absorbance values for representative desulfoglucosinolate solutions from *B. juncea*, *S. alba*, *R. sativus* and *E. sativa* extracts treated with sulfatase solution for 12, 24 or 48 h. In most cases, 12 and 24 h incubation periods were insufficient for complete desulfation of glucosinolates. Glucoraphenin decreased in all *R. sativus* leaf samples tested, from 24 to 48 h, while recovery of the internal standard increased, suggesting that specifically this desulfoglucosinolate is degraded during the purification process (Fig. 8).

**Fig. 9:**
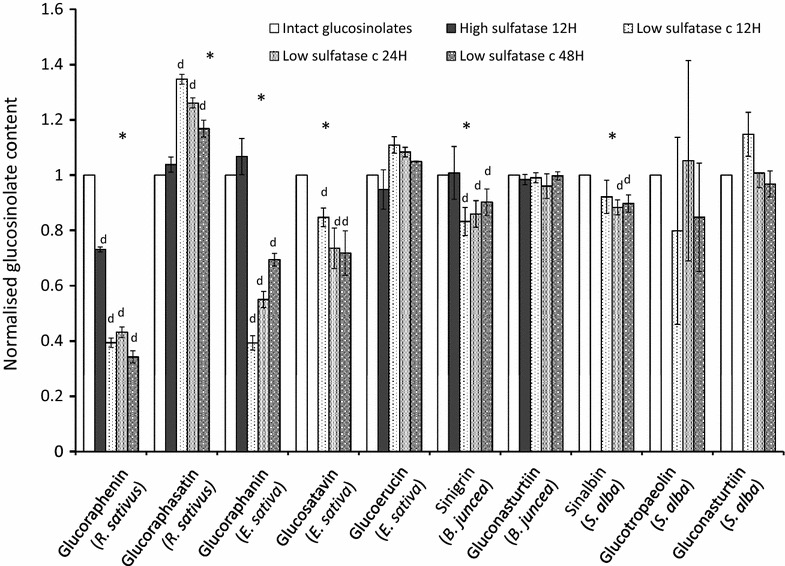
Desulfoglucosinolate content extracted from *B. juncea*, *E. sativa* and *R. sativus* tissue incubated with 75 µl low concentration sulfatase (0.3 U/ml) over 12, 24 or 48 h, and with a high concentration sulfatase (5 U/ml) over 24 h, normalised to glucosinolate content of the same samples prior to sulfatase treatment. *E. sativa* leaf samples were treated with TCEP post desulfation to undimerise didesulfoglucosatavin. Asterisks indicate a significant effect of purification method on glucosinolate concentration (repeat measure ANOVA, *p* < 0.05). ‘*d*’ indicates the purification method yields a significantly different glucosinolate concentration relative to the intact glucosinolates (paired *t* test, *p* < 0.05)

Not all glucosinolates are desulfated on the column at the same rate [31], meaning that incomplete desulfation of extractions is likely to yield imprecise results: overestimating or underestimating the final concentration of glucosinolates which are desulfated quicker or slower respectively than the internal standard. In addition, relative and total concentrations of glucosinolates and degradation or rearrangement of glucosinolates during this process can also affect final concentrations [26, 31]. Use of higher sulfatase concentrations than outlined in the ISO method has been suggested for glucosinolate analysis in *B. napus* and *B. oleracea* [25, 31]. Figure 9 compares relative glucosinolate concentrations from *B. juncea*, *S. alba*, *R. sativus* and *E. sativa* purified with a low activity sulfatase solution (0.3 U/ml) for 12, 24 and 48 h, a high activity sulfatase solution (5 U/ml) and intact glucosinolates. All concentrations have been normalised to the intact glucosinolate values. Desulfated glucosinolates concentrations obtained with high concentration sulfatase compared well with intact glucosinolates (Fig. 9). However, both high sulfatase as well as low sulfatase treatments yielded lower glucoraphenin content estimates. Coupled with the reduction of the recovery of desulfoglucoraphenin from 24 to 48 h (Fig. 8), these data suggest that glucoraphenin is degraded or transformed during the desulfation process.

Shorter desulfation times and lower sulfatase concentrations resulted in underestimation of the concentrations of glucoraphenin from *R. sativus*, glucoraphanin and glucosatavin from *E. sativa*, sinigrin from *B. juncea*, and sinalbin from *S. alba* and an overestimation of the concentrations of glucoraphasatin in *R. sativus* roots (Fig. 9). The overnight (12–24 h) incubation with 0.3 U/ml sulfatase solution yields inaccurate results for most major glucosinolates examined in this study. The ISO9167-1 method suggests that a diluted purified sulfatase solution with an activity exceeding 0.05 U/ml should be used, which is shown to be insufficient for glucosinolate analysis from plant samples and conditions examined in this study (Fig. 9). Instead, if a desulfation step is carried out, use of a higher concentration of purified sulfatase (in this case, 5 U/ml) is advised.

In all *E. sativa* leaf samples tested, recovery of monomeric desulfo-glucosatavin decreased and recovery of dimeric desulfo-glucosatavin increased between 24 and 48 h. Bennet et al. [22] previously hypothesised that dimeric glucosatavin is unlikely to be found in vivo and is probably an artefact of the extraction process. We can confirm that glucosatavin forms dimers as a result of the desulfation step of the extraction and that without carrying this step out and instead quantifying intact glucosinolates, no dimeric glucosatavin was detected in these samples.

Given that glucoraphenin concentration estimates are lower from methods employing a desulfation step, and that this step is also responsible for the dimerization of glucosatavin, analysis of intact glucosinolates is preferable in most instances. It is out of the scope of this study to compare or improve separation and detection methods but it should be noted that major glucosinolates in this study were accurately measured by a HPLC–UV method adapted from Herzallah and Holley [19]. For examination of low abundance glucosinolates, and to avoid any potential inaccuracies due to contamination it is advised that an alternative HPLC method such as those suggested in Lee et al. or Forster et al. be used instead [26, 32].

### Suggested method for glucosinolate extraction

#### Tissue disruption

Depending on whether freeze drying is required:

1. Freeze samples loosely wrapped in foil in liquid nitrogen and store at −80 °C. Transport samples to freeze drier in dry ice. Rapidly load samples onto a cool plate in freeze drier and ensure the pressure drops to below 5 mbar in under 2 min. Mill samples once dried and store in airtight containers in the dark.

or

1. Freeze 50 mg samples in liquid nitrogen in 2 ml eppendorf tubes and store at −80 °C (for larger samples use larger tubes). Add a volume of 80% methanol precooled to −20 °C ensuring that final methanol concentration remains above 78% according to Eq. (1) in materials and methods. Add an appropriate volume of internal standard sinigrin or glucotropaeolin (e.g. 100 µM final concentration). Disrupt tissue by adding 2 small ball bearings and agitating with a tissue lyser (e.g. tissuelyserII, Qiagen) for 10 min at 20 rev/s. Alternatively use a plastic pestle to thoroughly grind the sample taking care that to keep the media below 0 °C. Continue directly to 2b.

#### Extraction

2a For freeze dried tissue (1a). To 0.1 g tissue, add 5 ml of 80% methanol and 50 µL of 20 mM sinigrin solution. Then
2b Shake sample once and leave to stand for 30 min. Shake sample for a further 30 min (70 rev/s). Centrifuge at 4000 rpm and transfer supernatant to a fresh tube.

#### Desulfation

If desulfation is required, a high concentration sulfatase solution should be prepared by dissolving 15–25 mg sulfatase in 1 ml 40% ethanol and centrifuge at 8000 rmp for 1 min. Transfer supernatant to a fresh 2 ml eppendorf tube and add 1 ml of pure ethanol to precipitate the sulfatase and centrifuge at 8000 rpm for 1 min. Discard the supernatant and air dry the pellet before re-dissolving in 2 ml of water. Proceed with desulfation according to ISO9167-1 method.

## Conclusions

In this study we compared different methods for extracting and purifying glucosinolates from *B. napus*, *B. junea*, *S. alba*, *E. sativa* and *R. sativus* green tissues to highlight unnecessary or hazardous steps. We have presented a simplified method for extracting glucosinolates from plant tissues which does not require the use of a freeze drier or boiling methanol, and is therefore less hazardous, and more time and cost effective. The presented method has been shown to have comparable or improved glucosinolate extraction efficiency relative to the commonly used ISO method for major glucosinolates in the Brassicaceae species studied: sinigrin and gluconasturtiin in *B. juncea*; sinalbin, glucotropaeolin, and gluconasturtiin in *S. alba*; glucoraphenin and glucoraphasatin (roots but not shoots) in *R. sativus*; and glucosatavin, glucoerucin and glucoraphanin in *E. sativa*.

## Additional file

**Additional file 1.** Example chromatograms of desulfoglucosinolates and glucosinolates extracted according to methods described in this manuscript.
