# Supplementary material for: Development of an efficient glucosinolate extraction method
Source: Plant Methods. 2017 Mar 21;13:17. doi: 10.1186/s13007-017-0164-8 (PMC5361809; doi:10.1186/s13007-017-0164-8)
Supplement: Supplementary file 1 — Additional file 1. Example chromatograms of desulfoglucosinolates and glucosinolates extracted according to methods described in this manuscript. [file 13007_2017_164_MOESM1_ESM.docx]

Example Chromatograms

# Chromatograms of desulfo-glucosinolates

Fig S1: Example chromatograms from *E. sativa* leaf (left) and root (right) extractions following desulfation. Desulfoglucosinolates: Sinigrin (8.6 min), Glucoraphanin (10.4 min) Glucosatavin (16.6 min), Glucoerucin (19.5 min).


Fig S2: Example chromatograms from *R. sativus* leaf (left) and root (right) extractions following desulfation. Desulfoglucosinolates: Sinigrin (8.5-8.7 min), Glucoraphenin (11-11.7 min) and Glucoraphasatin (19.8 min).


Fig S3: Example chromatograms from *B. juncea* leaf (left) and root (right) extractions following desulfation. Desulfoglucosinolates: Sinigrin (8.5 min), Glucotropaeolin (19 min), Glucobrassicin (21.4 min) and Gluconasturtiin (23.2 min). Unidentified glucosinolates (16.5 min, 28 min)


Fig S4: Example chromatograms from *S. alba* leaf (left) and root (right) extractions following desulfation. Desulfoglucosinolates: Sinigrin (8.6 min), Sinalbin (13.6 min), Glucotropaeolin (18.8-19 min), Glucoerucin (20 min) and Gluconasturtiin (23.6 min), Methoxyglucobrassicin (28.4 min). Unidentified glucosinolate (24 min).

# Chromatograms of intact glucosinolates (no desulfation step)

Fig S5: Example chromatograms from *B. juncea* leaf (left) and root (right) extractions without purification. Glucosinolates: Sinigrin (11.4-11.8 min), Glucobrassicin (23.8 min), Gluconasturtiin (27.5-28.5min).

Fig S6: Example chromatograms from *S. alba* leaf (left) and root (right) extractions without purification. Glucosinolates: Sinigrin (11.1), Sinalbin (16.4-16.6 min), Glucotropaeolin (23.7 min) Gluconasturtiin (28.7 min).

Fig S7: Example chromatograms from *E. sativa* leaf (left) and root (right) extractions without purification. Glucosinolates: Glucoraphanin (5.6 min), Sinigrin (11.5-12.1 min), Glucosatavin (22.9 min) and Glucoerucin (25.4 min).

Fig S8: Example chromatograms from *R. sativus* leaf (left) and root (right) extractions without purification. Glucosinolates: Glucoraphenin (5.8-6.2 min), Sinigrin (11.5-11.9 min), Glucoraphasatin (25.7-26.4 min).
